# Total Metabolic Syndrome Score in Addition To FIB-4 Score May Be Useful in Predicting Advanced Fibrosis in Metabolic Dysfunction-Associated Steatotic Liver Disease

**DOI:** 10.5152/tjg.2025.25058

**Published:** 2025-05-20

**Authors:** Burcu Gurbuz, Bahadir Koylu, Onur Keskin

**Affiliations:** 1 Department of Gastroenterology, Hacettepe University Faculty of Medicine, Ankara, Türkiye; 2 Department of Medical Oncology, Koç University Faculty of Medicine, İstanbul, Türkiye

Dear Editor,

The study by Şahintürk et al^1^ investigating the prevalence of metabolic dysfunction-associated steatotic liver disease (MASLD) in type 2 diabetes mellitus patients and the prediction of advanced fibrosis using the FIB-4 score in patients diagnosed with MASLD was read with great interest. The authors are commended for their work on raising awareness about MASLD among internal medicine specialists. According to their findings, approximately 30% of patients with type 2 diabetes underwent ultrasonography for various reasons, with 70% of these patients showing hepatic steatosis. In diabetic patients diagnosed with MASLD by ultrasonography, the FIB-4 score was calculated as a non-invasive fibrosis marker, and 24.6% of patients had scores above the specified cut-off value, suggesting advanced fibrosis.

The article indicates that only 52 (17.9%) of the patients with suspected advanced fibrosis were referred to the gastroenterology department. It is not clear from the article whether the FIB-4 calculation was made during the patient’s examination or whether it was done retrospectively in MASLD patients. If advanced fibrosis suspicion was detected with the FIB-4 calculation during the patient’s examination and a gastroenterology referral was not made despite this, it indicates that the awareness of the importance of MASLD among internal medicine specialists is quite low. Internal medicine specialists should be reminded more frequently that metabolic dysfunction associated steatohepatitis (MASH) is a progressive liver disease and is also an important cause of cirrhosis in Türkiye.^2^ Disease progression can be prevented with simple lifestyle changes in MASLD. Some simple parameters that internal medicine specialists inquire about in every examination, in addition to USG (ultrasonography) and FIB-4, can also be guiding.

In a study of biopsy-proven MASLD patients, the presence of diabetes mellitus, hypertension, dyslipidemia, and metabolic syndrome criteria were evaluated. Patients were scored with 1 point for each of the 5 diagnostic criteria of metabolic syndrome to calculate a total metabolic syndrome score. According to liver biopsy results, metabolic syndrome was present in 47.8% of patients with ≤F1 fibrosis and 84.8% of patients with ≥F2 fibrosis (*P*: .001). Additionally, 60.6% of patients with ≥F2 fibrosis had a total metabolic syndrome score of 4 or higher. Correlation analysis revealed a significant correlation between total metabolic syndrome score and fibrosis stage (r: 0.48, *P *< .001).^3^ These findings underscore the importance of comprehensive metabolic evaluation in risk stratification for MASLD patients. Indeed, previous studies have shown that metabolic syndrome criteria can be an independent predictor of mortality in chronic liver diseases.^4^

Waist circumference, presence of hypertension, blood glucose tests, and serum lipid levels are parameters routinely evaluated by internal medicine specialists. The combined use of the easily calculated FIB-4 score, particularly in patients with high total metabolic scores, could facilitate the identification of high-risk patients for fibrosis. Referral of these patients to gastroenterologists for further evaluation would enable early detection of advanced fibrosis. Currently, non-invasive tests such as MR elastography and transient elastography for detecting advanced fibrosis in MASH patients are available in some tertiary centers. While liver biopsy remains the definitive test for diagnosing steatohepatitis and excluding alternative liver diseases, it is not required in most cases for the clinical management of MASLD patients.^5^

In conclusion, the combined use of the total metabolic syndrome score and FIB-4 in primary and secondary healthcare settings could improve the identification of patients at risk for advanced fibrosis. The coexistence of multiple metabolic syndrome components may accelerate fibrosis progression, highlighting the importance of evaluating not only diabetes but all metabolic syndrome criteria. This comprehensive approach to risk assessment, incorporating both metabolic and fibrosis parameters, may help optimize the care pathway for MASLD patients and potentially improve outcomes through earlier intervention.

## Data Availability

The data that support the findings of this study are available upon request from the corresponding author.
